# A Japanese herbal medicine (kampo), hochuekkito (TJ-41), has anti-inflammatory effects on the chronic obstructive pulmonary disease mouse model

**DOI:** 10.1038/s41598-024-60646-x

**Published:** 2024-05-06

**Authors:** Masaaki Yuki, Taro Ishimori, Shiho Kono, Saki Nagoshi, Minako Saito, Hideaki Isago, Hiroyuki Tamiya, Kensuke Fukuda, Naoya Miyashita, Takashi Ishii, Hirotaka Matsuzaki, Yoshihisa Hiraishi, Akira Saito, Taisuke Jo, Takahide Nagase, Akihisa Mitani

**Affiliations:** 1 Department of Respiratory Medicine, The University of Tokyo, 7-3-1 Hongo, Bunkyo-Ku, Tokyo, 113-8655 Japan; 2 Department of Clinical Laboratory Medicine, Graduate School of Medicine, The University of Tokyo Hospital, 7-3-1 Hongo, Bunkyo-Ku, Tokyo, 113-8655 Japan; 3 Division for Health Service Promotion, The University of Tokyo, 7-3-1 Hongo, Bunkyo-Ku, Tokyo, 113-0033 Japan; 4 Center for Epidemiology and Preventive Medicine, The University of Tokyo Hospital, 7-3-1 Hongo, Bunkyo-Ku, Tokyo, 113-8655 Japan; 5 Department of Health Services Research, The University of Tokyo, 7-3-1 Hongo, Bunkyo-Ku, Tokyo, 113-8655 Japan

**Keywords:** Cell biology, Drug discovery, Diseases, Medical research

## Abstract

Chronic obstructive pulmonary disease (COPD) is a progressive disease that is characterized by chronic airway inflammation. A Japanese herbal medicine, hochuekkito (TJ-41), is prominently used for chronic inflammatory diseases in Japan. This study aimed to analyze the anti-inflammatory effect of TJ-41 in vivo and its underlying mechanisms. We created a COPD mouse model using intratracheal administration of porcine pancreatic elastase and lipopolysaccharide (LPS) and analyzed them with and without TJ-41 administration. A TJ-41-containing diet reduced inflammatory cell infiltration of the lungs in the acute and chronic phases and body weight loss in the acute phase. In vitro experiments revealed that TJ-41 treatment suppressed the LPS-induced inflammatory cytokines in BEAS-2B cells. Furthermore, TJ-41 administration activated the AMP-activated protein kinase (AMPK) pathway and inhibited the mechanistic target of the rapamycin (mTOR) pathway, both in cellular and mouse experiments. We concluded that TJ-41 administration reduced airway inflammation in the COPD mouse model, which might be regulated by the activated AMPK pathway, and inhibited the mTOR pathway.

Chronic obstructive pulmonary disease (COPD) is a progressive disease that is characterized by progressive airway obstruction, which is associated with abnormal airway inflammation caused by noxious particles^1^. In recent years, COPD has been regarded as a systemic inflammatory disease, not limited to the lungs. Generally, inflammatory cytokines, such as C-reactive protein (CRP), interleukin (IL)-6, and IL-8, are increased in the serum of patients with COPD^2^, and they are accompanied by weight loss and muscle weakness^3,4^. Approximately 25% of patients with COPD are affected by weight loss and malnutrition^3^. Patients with COPD are prone to sarcopenia due to reduced physical activity and malnutrition^5^. The management goal included improvement in both symptoms and quality of life.

The pathogenesis of COPD is closely associated with amplified inflammation, increased reactive oxygen species (ROS) production due to increased oxidants derived from inflammatory cells, and protease activation derived from inflammatory cells^6^. AMP-activated protein kinase (AMPK) pathway activation and mechanistic target of the rapamycin (mTOR) pathway inhibition have been reported as factors that regulate ROS^7^ and autophagy, which are known to affect the muscle mass of patients with COPD^8,9^. Several studies have reported the anti-inflammatory effects of the activated AMPK pathway in COPD pathogenesis^10,11^.

A Japanese herbal medicine (kampo), hochuekkito (TJ-41), is well-known in China, Japan, and South Korea^12^. TJ-41 is prevalently used for chronic disease or weakness after illness, with the expectation of improving gastrointestinal and autonomic nervous system functions^13,14^. TJ-41 consists of 10 herbal medicines, including *Astragali radix* (16.7%), *Atractyloclis lancea rhizome* (16.7%), *Ginseng radix* (16.7%), *Angelicae radix* (12.5%), *Bupleuri radix* (8.3%), *Zizyphi fructus* (8.3%), *Aurantii nobilis pericarpium* (8.3%), *Glycyrrhizae radix* (6.3%), *Cimicifugae rhizoma* (4.2%), and *Zingiberis rhizoma* (2.0%). Several TJ-41 ingredients, including *Astragali radix*^15,16^, *Ginseng radix*^17^, *Angelicase radix*^18^, and *Bupleuri radix*^19,20^, reportedly have anti-inflammatory effects.

Several studies reported the effect of TJ-41 when administered to patients with COPD^21–24^. TJ-41 treatment reduced weight loss, COPD exacerbations, serum CRP, tumor necrosis factor-alpha (TNF-α), and IL-6 levels, improved COPD assessment test scores, and enhanced quality of life in patients with COPD^21–23^. Furthermore, our previous report revealed that TJ-41 suppressed LPS-induced inflammation in a genetically engineered mouse model of emphysema^24^. However, the biological mechanisms of the anti-inflammatory effect of TJ-41 and the improvement of the systemic conditions of patients with COPD remained unclear.

A COPD mouse model was developed through the intratracheal administration of porcine pancreatic elastase (PPE) and lipopolysaccharide (LPS)^25^. Intratracheal PPE administration causes lung emphysema^26,27^, whereas LPS administration after PPE exacerbates inflammatory cell infiltration within a few weeks^28^. This method is commonly used to study the COPD mouse model. However, no studies have yet reported the effects of TJ-41 on a COPD mouse model created using this method.

This study aimed to determine the anti-inflammatory and systemic effects of TJ-41 on the PPE- and LPS-induced COPD mouse model and investigate its underlying mechanism on the anti-inflammatory effect.

## Results

### TJ-41 attenuates inflammatory cell infiltration of the emphysematous lungs

**Figure 1:**
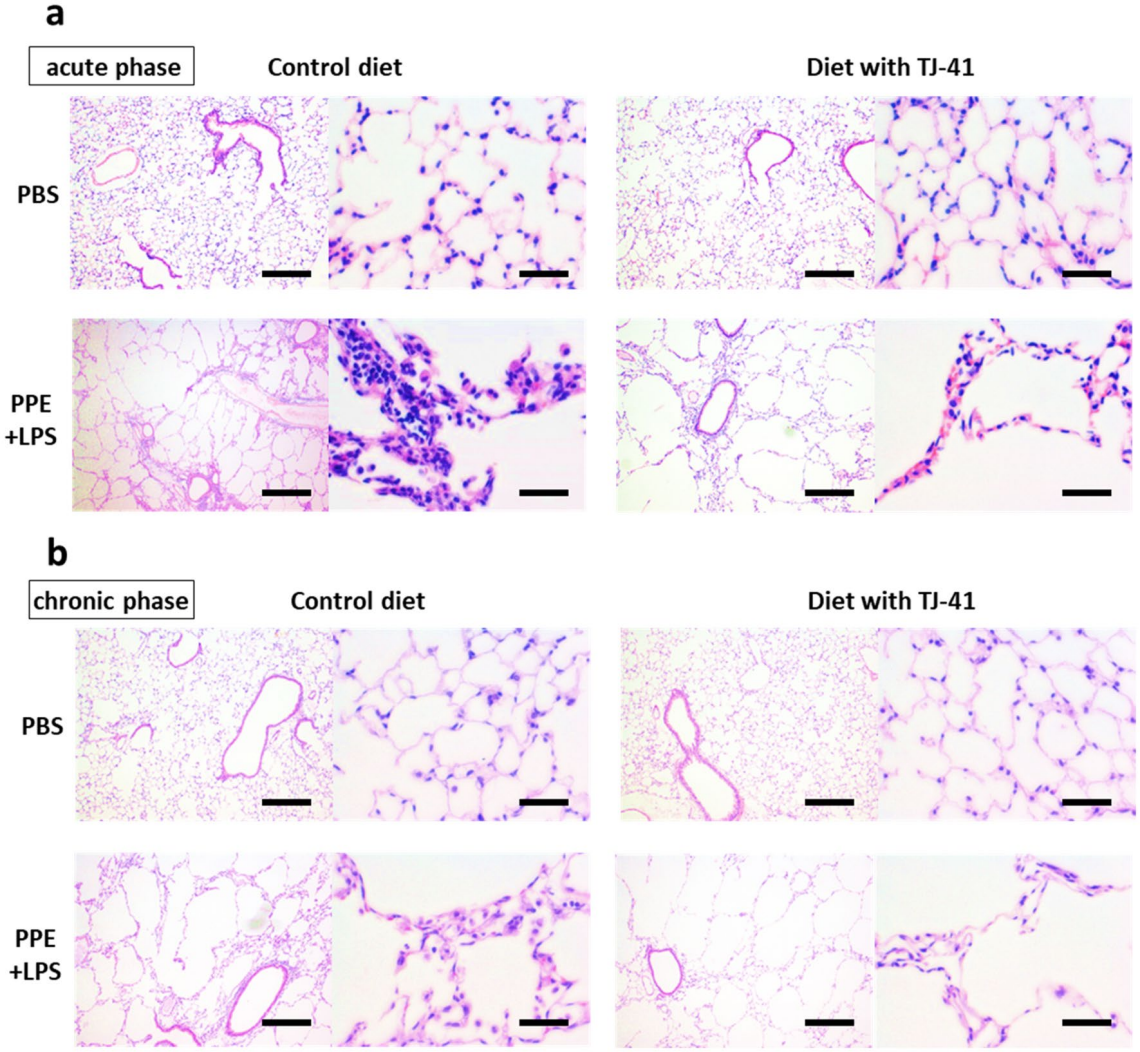
Hematoxylin–eosin staining of the lungs of COPD mice models with or without TJ-41 treatment in the acute phase (**a**) and the chronic phase (**b**). Magnification in the left image, × 40; magnification in the right image, × 200. The scale bar showed 250 µm in the left image and 50 µm in the right image.

Histological studies revealed that mice lungs treated with PPE and LPS showed emphysematous changes and inflammatory cell infiltration in the acute phase (Fig. 1a). A TJ-41-containing diet reduced inflammatory cell infiltration of the lungs, although it did not improve emphysematous changes. The mouse model in the chronic phase demonstrated worsened emphysematous change and reduced inflammatory cell infiltration, compared to the acute phase (Fig. 1b). Further, the TJ-41-containing diet reduced inflammatory cell infiltration of the lungs in the chronic phase but did not affect emphysematous changes.

**Figure 2:**
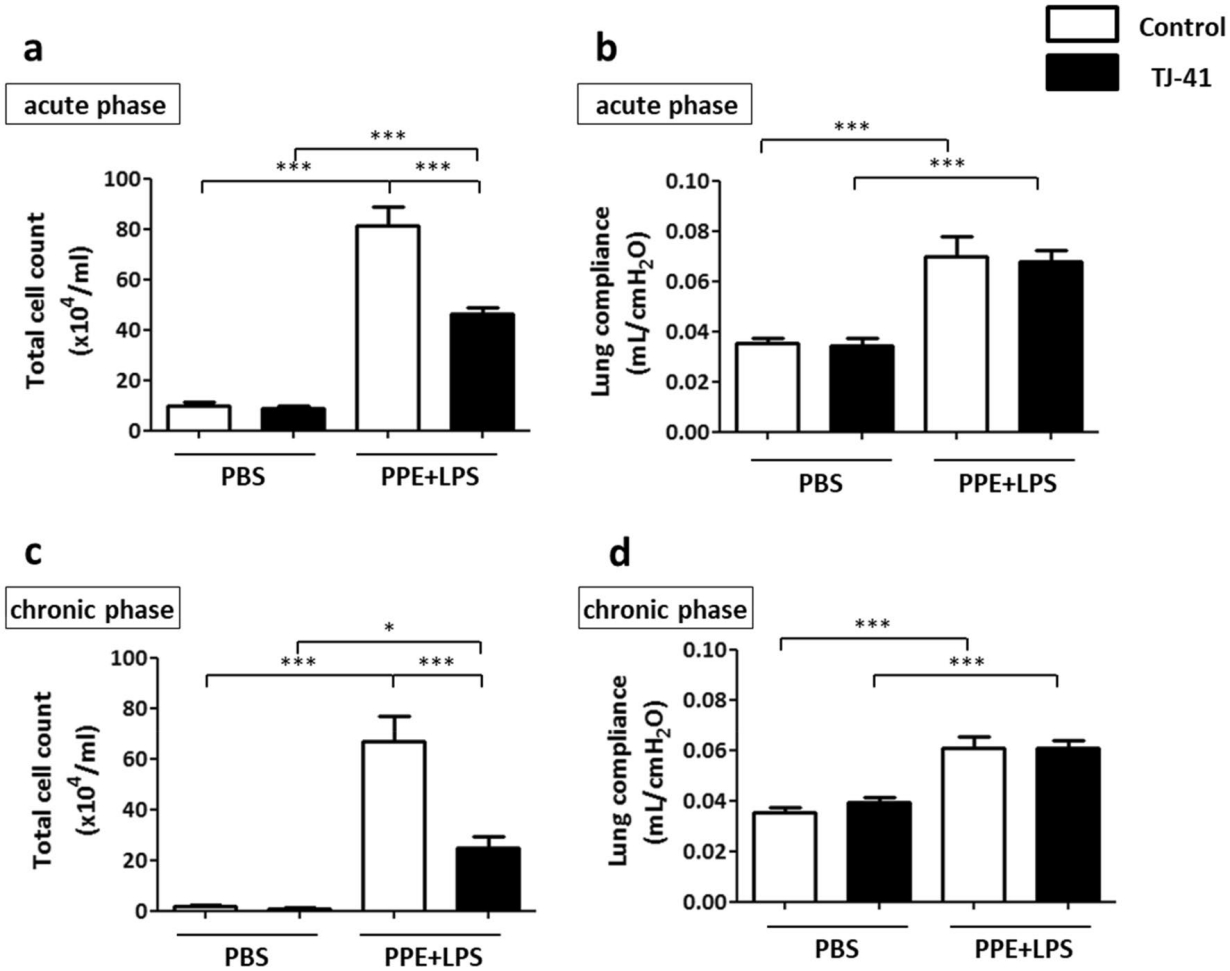
BALF analysis in the acute (**a**) and chronic (**c**) phases revealed that TJ-41 administration mitigated the PPE- and LPS-induced increase in pulmonary inflammatory cells; N = 3–5 per group. (**b** and **d**) Lung compliance was increased by PPE and LPS administration in both phases. One-way analysis of variance and Tukey’s multiple comparison tests with *P < 0.05, **P < 0.01, and ***P < 0.001 were considered statistically significant; N = 7–9 in each group.

The number of total cells in BALF was markedly higher in mice treated with PPE and LPS than in mice treated with phosphate-buffered saline (PBS), compatible with the histological analysis (Fig. 2a, c). The number of total cells in BALF of TJ-41-fed mice was significantly decreased compared to that of control diet-fed mice among the COPD mice model, both in the acute phase (8.1 ± 0.8 × 10^5^ /mL vs. 4.6 ± 0.3 × 10^5^ /mL; *p* < 0.001) and in the chronic phase (6.7 ± 1.0 × 10^5^ /mL vs. 2.5 ± 0.5 × 10^5^ /mL; *p* < 0.001) (Fig. 2a, c). Inflammatory cells were predominantly composed of macrophages, although with a few percentages of neutrophils and lymphocytes. The numbers of macrophages in BALF of TJ-41-fed mice treated with PPE and LPS were significantly decreased compared to those of control diet-fed mice, both in the acute phase (7.8 ± 0.7 × 10^5^ /mL vs. 4.4 ± 0.3 × 10^5^ /mL; *p* < 0.001) and in the chronic phase (6.3 ± 1.0 × 10^5^ /mL vs. 2.3 ± 0.4 × 10^5^ /mL; *p* < 0.001) (Supplementary Fig. S1a, b).

We then measured lung physiological functions, and the COPD mice model demonstrated a significantly increased lung compliance compared to PBS-treated mice, both in the acute phase (0.035 ± 0.002 mL/cmH_2_O vs. 0.070 ± 0.008 mL/cmH_2_O; *p* < 0.001) and in the chronic phase (0.035 ± 0.002 mL/cmH_2_O vs. 0.061 ± 0.004 mL/cmH_2_O; *p* < 0.001). The physiological function with or without TJ-41 administration demonstrated no changes (Fig. 2b, d).

### TJ-41 inhibits body weight loss after PPE and LPS administration

**Figure 3:**
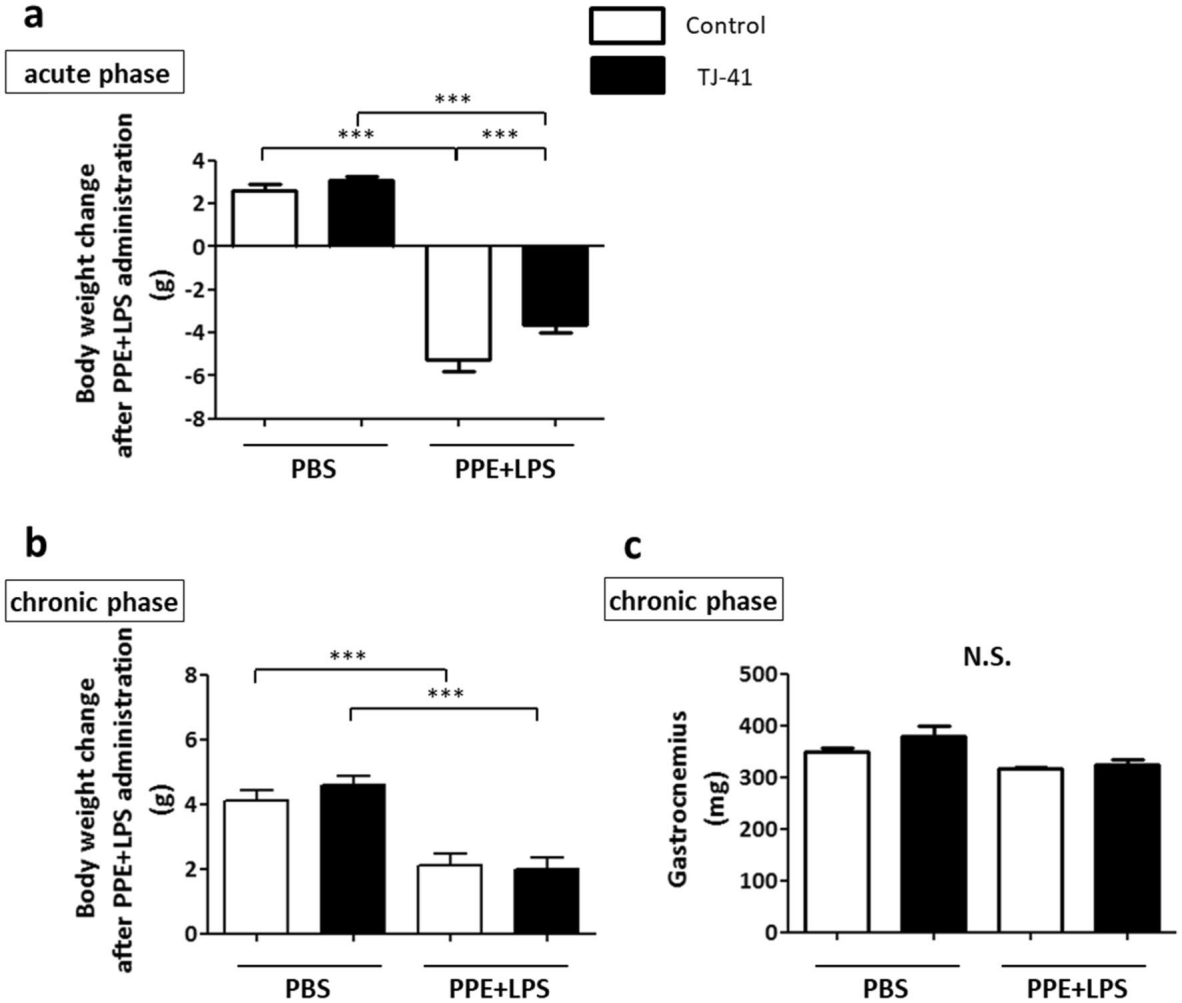
(a and b) The change in body weight at day 24 and day 49 in the acute (**a**) and chronic phases (**b**), respectively. (**c**) The weight of the gastrocnemius muscle at day 49; N = 7–9 in each group. One-way analysis of variance and Tukey’s multiple comparison tests with *P < 0.05 and ***P < 0.001 were considered statistically significant. *N.S*.: Not significant.

The COPD mice model lost body weight just after the last LPS dose. After 3 days, at day 24, the body weight loss reached the maximum, and it recovered gradually afterward (data not shown). At that point, the maximum weight loss of TJ-41-treated mice in the PPE and LPS groups was suppressed compared to control-treated mice (− 5.3 ± 0.5 g vs. − 3.6 ± 0.4 g ; *p* < 0.05) (Fig. 3a). At day 49, weight change demonstrated no difference with or without TJ-41 (Fig. 3b) neither the weight of the gastrocnemius muscle (Fig. 3c).

### TJ-41 administration attenuates LPS-induced IL-8 and TNF-alpha expression in human epithelial cells in vitro

The results of the in vivo experiments using the COPD mice model indicated that TJ-41 has a protective role on airway inflammation. To reveal the underlying mechanisms, in vitro studies are performed using BEAS-2B cells, which are human epithelial cell lines, and U937 cells, which are human monocyte cell lines.

**Figure 4:**
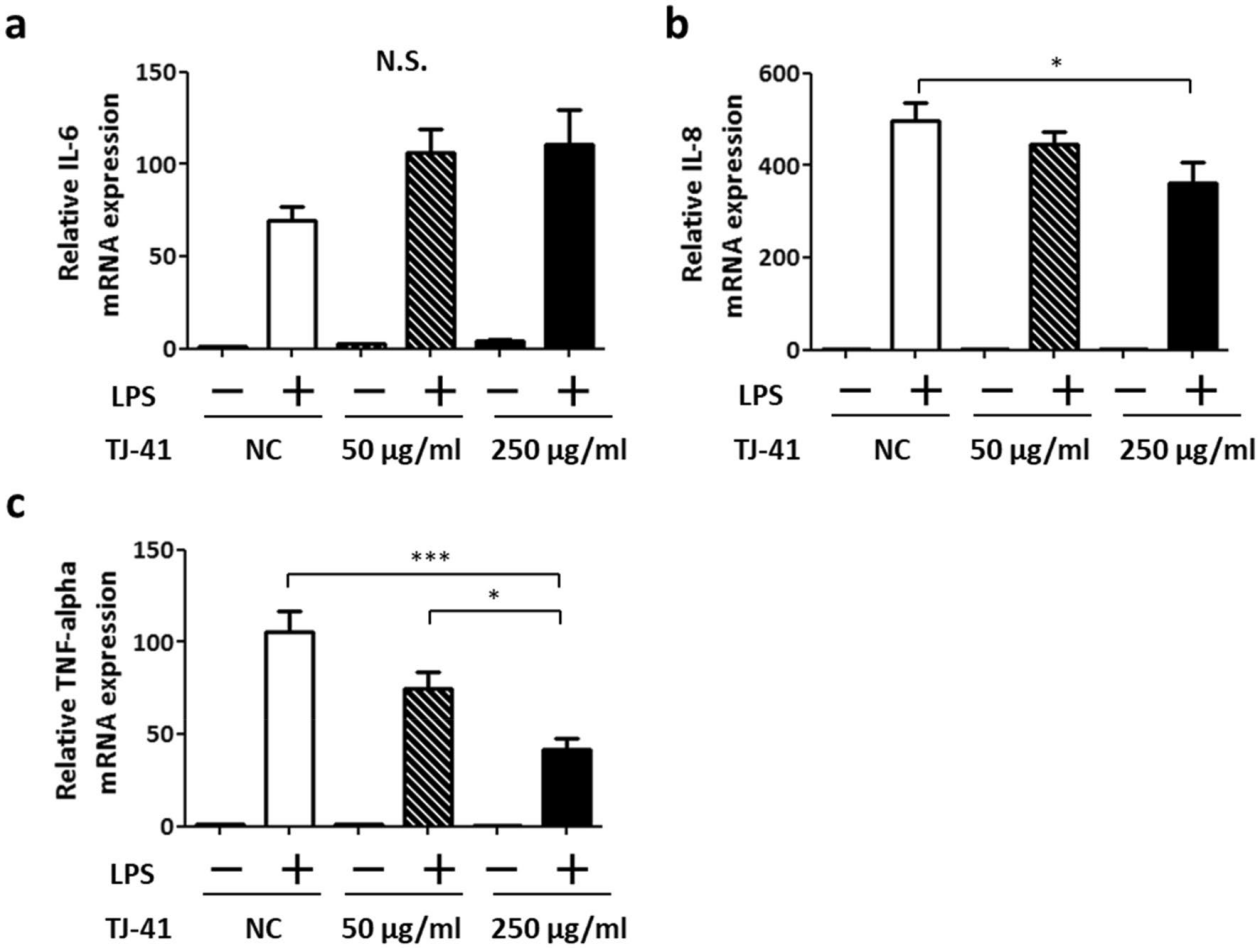
mRNA expressions of inflammatory cytokines, IL-6 (**a**), IL-8 (**b**), and TNF-α (**c**), in BEAS-2B cells after LPS treatment with or without TJ-41 administration. One-way analysis of variance and Tukey’s multiple comparison tests were performed in the three groups treated with LPS, and *P < 0.05 and ***P < 0.001 were considered statistically significant; N = 10 in each group. *NC*: negative control; *N.S*.: Not significant.

LPS stimulation induced the mRNA expressions of IL-6, IL-8, and TNF-α in BEAS-2B cells (Fig. 4). LPS-induced IL-8 expression and TNF-alpha expression were significantly decreased with TJ-41 treatment (Fig. 4b, c).

The expressions of IL-8 and TNF-α mRNAs were significantly elevated by LPS stimulation in U-937 cells (Supplementary Fig. S2b, c). TJ-41 treatment slightly reduced LPS-induced TNF-α expression, but without statistical significance (Supplementary Fig. S2c).

### TJ-41 activates the AMPK pathway and inhibits the mTOR pathway

The AMPK pathway activation and the mTOR pathway inhibition have been reported as factors that regulate ROS, which are implicated in inflammation in COPD, thus we investigated the association of TJ-41 with these pathways in BEAS-2B cells and U937 cells.

**Figure 5:**
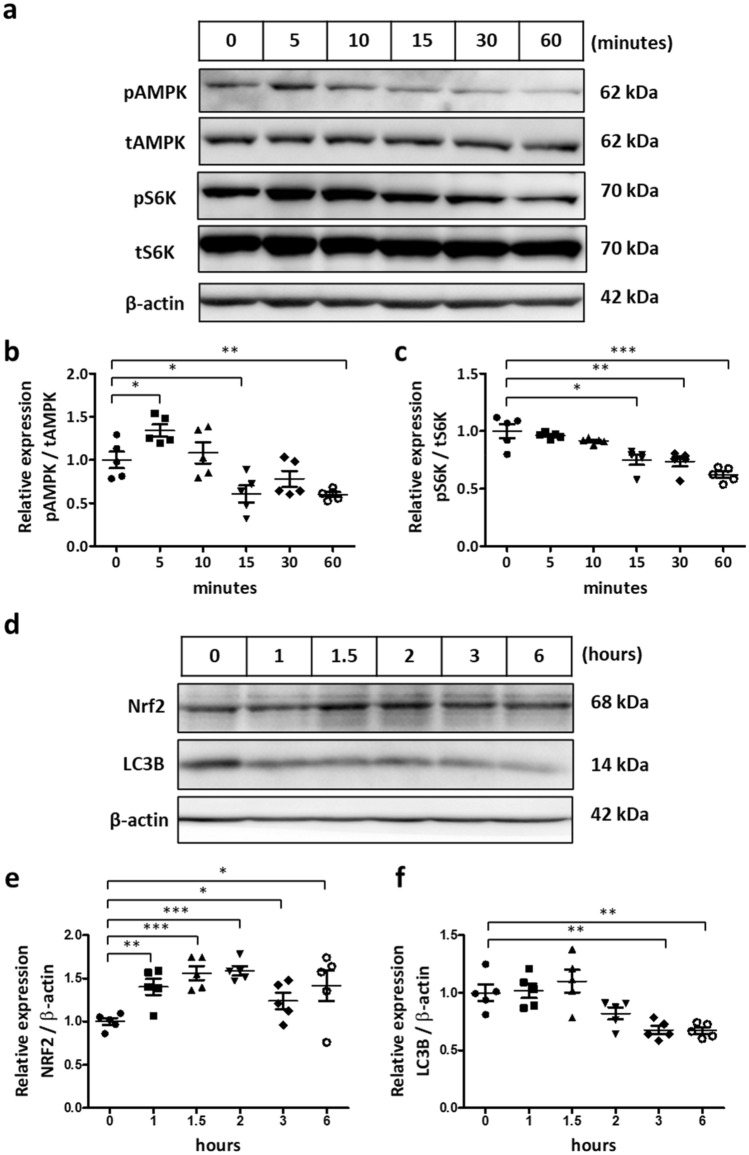
(**a**–**c**) Western blotting analysis of BEAS-2B cells from 5 to 60 min after TJ-41 treatment. (**d**–**f**) Long-time analysis from 1 to 6 h of TJ-41-treated BEAS-2B cells. Unpaired *t*-tests were used for each antibody, and *P < 0.05, **P < 0.01, and ***P < 0.001 were considered statistically significant. N = 5 in each group.

Time course analysis between 5 and 60 min in BEAS-2B cells revealed a significantly increased AMPK activation at 5 min after TJ-41 administration (Fig. 5a, b). Conversely, mTOR activation, represented by pS6K/tS6k, was significantly reduced after 15 min (Fig. 5a, c). Long-term analysis of up to 6 h revealed that the Nrf2 expression reached its peak at 1.5–2 h (Fig. 5d, e), whereas LC3B started to decrease at 3 h (Fig. 5d, f). Original blots/gels were presented in Supplementary Figures S3, S4.

The AMPK activation increased significantly at 5 min after TJ-41 administration in U-937 cells, as well as in BEAS-2B cells (Supplementary Fig. S5a, b). Conversely, mTOR activation was significantly reduced after 60 min (Supplementary Fig. S5a, c). The Nrf2 expression also reached its peak at 1.5–2 h after TJ-41 administration (Supplementary Fig. S5d, e). Unlike BEAS-2B cells, LC3B increased at 1.5–2 h in U-937 cells (Supplementary Fig. S5d, f). Original blots/gels were presented in Supplementary Figures S6, S7.

### Lung tissues from TJ-41-fed mice demonstrated an activated AMPK pathway and decreased mTOR pathway

**Figure 6:**
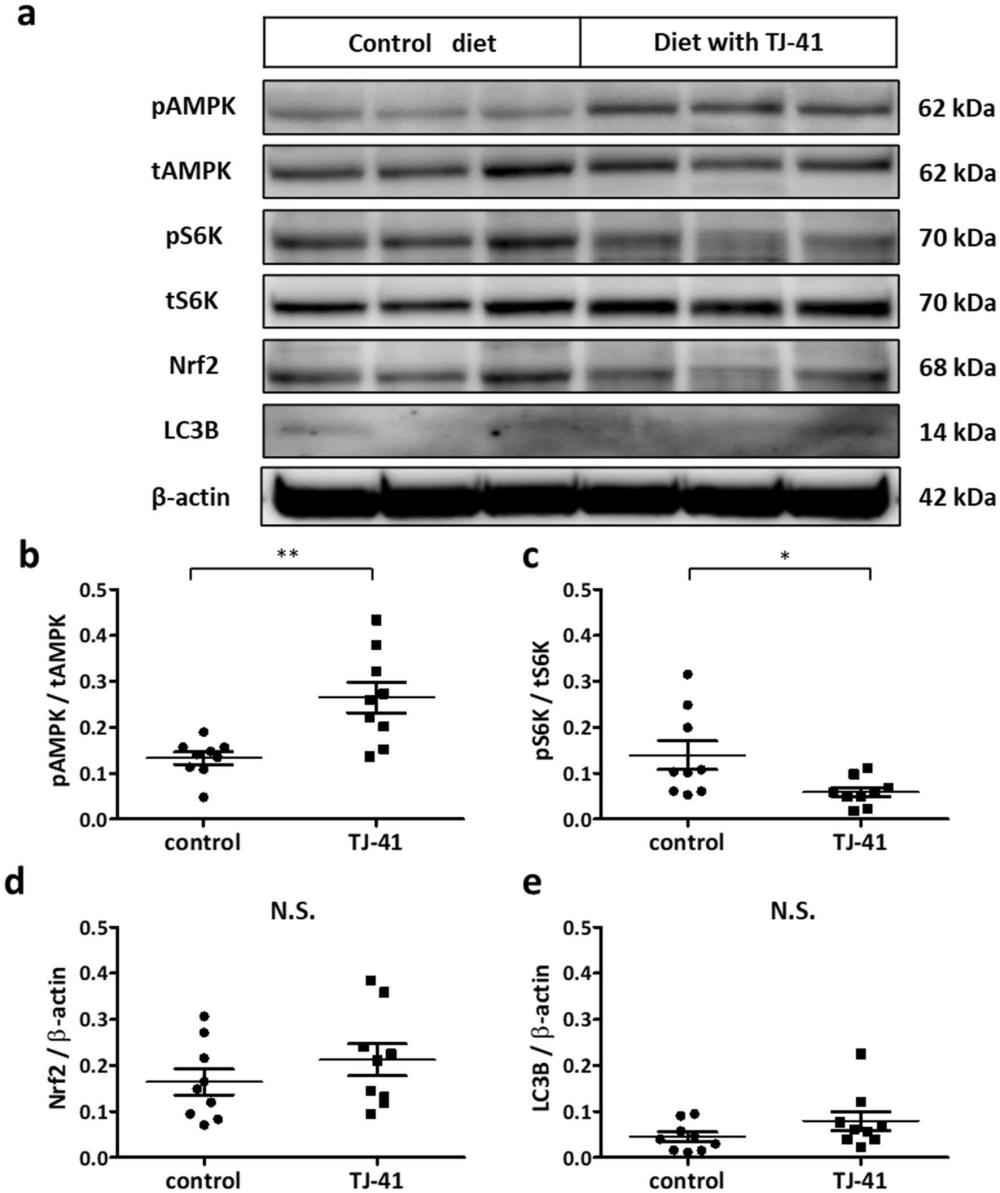
(**a**–**e**) Western blotting analysis using the lung tissues from wild-type mice that had been fed a TJ-41-containing diet. AMPK activity (b), mTOR activity (**c**), Nrf2 expression (**d**), and LC3B expression (**e**) were measured. Unpaired *t*-tests with **P < 0.01 were considered statistically significant for each antibody. N = 6 in each group. *N.S*.: not significant.

We extracted proteins from the whole lung tissues of mice fed a control or TJ-41-containing diet from 8 to 17 weeks of age. The lung tissues from mice fed a TJ-41-containing diet for a long period showed demonstrated AMPK activation and decreased mTOR activation compared to the mice fed a control diet (Fig. 6a, b, c). The expressions of Nrf2 and LC3B demonstrated no change with or without TJ-41 (Fig. 6a, d, e). Original blots/gels were presented in Supplementary Figure S8.

### RNA sequencing shows that neutrophilic inflammation is altered by PPE and LPS administration in mice

Total RNA was collected from the lung tissues of the COPD mice model and the control mice and was analyzed by RNA sequencing.

**Figure 7:**
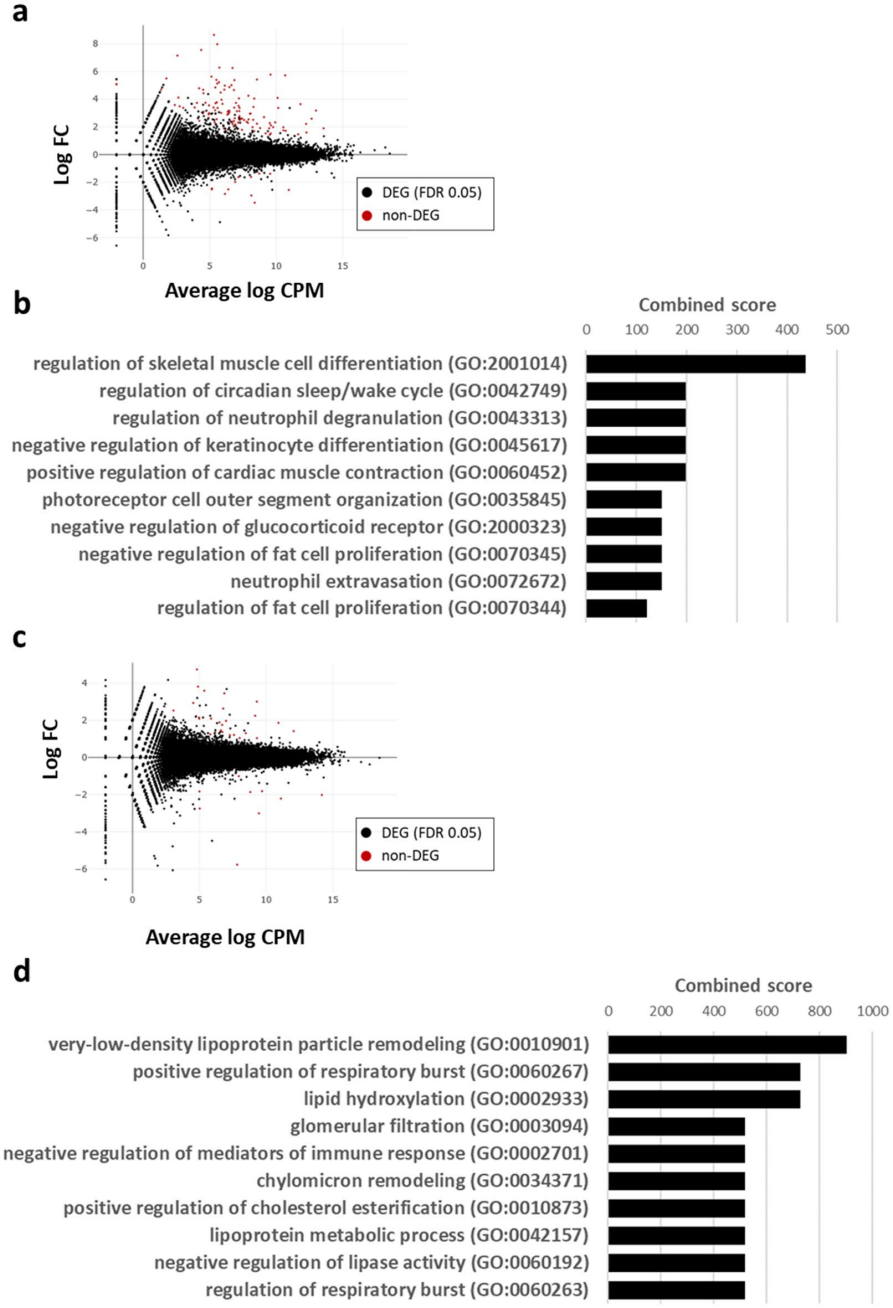
RNA sequencing analysis. (**a**) MA plot showing the RNA expression profile between control mice and COPD mice model. (**b**) The top 10 enriched biological process gene ontology terms are sorted by Enrichr combined score. (**c**) MA plot showing the RNA expression profile between the presence and absence of TJ-41 administration in the control mice. (**d**) The top 10 enriched biological process gene ontology terms. *CPM*: Counts per million; *DEG*: Differential expression gene; *FC*: Fold change; *FDR*: False discovery rate; *GO*: Gene ontology.

First, we defined differential expression genes (DEG) between control mice and COPD mice model (Fig. 7a). We conducted gene ontology analysis of the DEG, focusing on the biological process category of terms (Fig. 7b), and terms related to neutrophil degranulation and neutrophil extravasation were detected, consistent with the in vivo experiments.

We then defined DEGs between the presence and absence of TJ-41 administration in the control mice (Fig. 7c). The gene ontology analysis of the DEG, focusing on the biological process category of terms (Fig. 7d), detected terms related to positive regulation of respiratory burst. This indicates the lungs from TJ-41-fed mice demonstrated a rapid increase in the metabolic activity of neutrophils, monocytes, and other phagocytes, which is associated with phagocytosis.

## Discussion

### Anti-inflammatory effects of TJ-41 on COPD model lungs

The pathological studies and BALF analyses demonstrated the anti-inflammatory effect of oral TJ-41 administration in the lungs of the COPD mice model. TJ-41, particularly, suppressed macrophage and neutrophilic inflammation in the airspace. These results are compatible with the previous human studies in which TJ-41 treatment decreased the expression level of inflammatory cytokines in patients with COPD^23^.

On the other hand, TJ-41 administration had little effect in pathological and physiological studies in mice, as for emphysematous changes, the same as reported in human studies^23^. However, because repeated exacerbations in COPD supposedly cause emphysematous changes in the mouse model with repeated stimulation, TJ-41 might affect emphysematous changes by suppressing inflammation. Further investigation is required.

TJ-41 treatment reportedly improves body weight loss in patients with COPD^21^. In our COPD mouse model, acute phase TJ-41 also reduced weight loss. Furthermore, white fat levels reportedly decrease in mice after PPE administration^29^, which could cause weight loss. It is also likely related to various other factors associated with chronic inflammation, including decreased appetite and dehydration.

In vitro studies, TJ-41 suppressed the LPS-induced inflammatory cytokines in BEAS-2B cells, consistent with the results of the mouse experiments. These results were also consistent with human studies demonstrating lowered serum inflammatory markers in patients with COPD with TJ-41 administration. The number of alveolar macrophages increased significantly in the airways of patients with COPD^30^, and alveolar macrophages secrete various inflammatory cytokines^31^, contributing to COPD pathogenesis. Therefore, we analyzed the effects of TJ-41 on monocyte-derived U937 cells. However, in this study, no anti-inflammatory effect was observed.

### TJ-41 herbal ingredients

In this study, we investigated the anti-inflammatory effects of TJ-41. Some of its components, including astragalin and astragaloside IV in *Astragali radix*^15,16^, ginsenoside Rb1 in *Ginseng radix*^*17*^, ferulic acid in *Angelicase radix*^18^, and saikosaponin A in *Bupleuri radix*^19,20^, reportedly have anti-inflammatory effects. Given that TJ-41 is a traditional combination of these natural herbal medicines and each component is not taken separately, we analyzed the total anti-inflammatory effect of TJ-41.

### TJ-41 mediated AMPK pathway activation and mTOR inhibition

Our in vitro experiments using BEAS-2B cells and U937 cells revealed that TJ-41 administration activated the AMPK pathway and inhibited the mTOR pathway. Time course analysis, particularly, indicated that TJ-41 administration first activated AMPK followed by the mTOR pathway inhibition. Further, in vivo studies revealed that long-term TJ-41 administration caused activated AMPK pathway and reduced mTOR pathway in mouse lungs. This is the first study to demonstrate the intracellular effect of TJ-41 treatment. Several reports have revealed that AMPK activation has an anti-inflammatory effect on patients and mouse models with COPD^9,32^, indicating that TJ-41 might cause an anti-inflammatory effect via AMPK activation.

Moreover, TJ-41 altered Nrf2 and LC3B expressions, indicating that TJ-41 may affect autophagy. TJ-41 might be involved in stress regulation, such as oxidative stress, by driving autophagy in the COPD model. Further investigation is required.

### Limitations of this study

Our study has some limitations. First, in vitro studies administered TJ-41 directly to BEAS-2B cells and U937 cells to investigate its effects. However, TJ-41 is taken orally and metabolized in the body before affecting the human body. Therefore, the results of our in vitro studies reveal that TJ-41 activates the AMPK pathway and inhibits the mTOR pathway and may not accurately represent the effects of TJ-41 when metabolized in the human body, although our in vivo study revealed the increased AMPK activation in the lungs from TJ-41-fed mice.

Second, the lungs of the COPD mice model induced by intratracheal administration of PPE and LPS in this experiment are not the same as the lungs of patients with COPD, although the COPD mice model using PPE and LPS is one of the most prominent models. For example, the cigarette smoke-induced COPD mouse model requires a much longer period compared with our model, and the emphysematous changes are rather subtle. Therefore, the possibility that this model is more suitable for detecting the effect of TJ-41 on emphysematous change or body weight loss cannot be ruled out.

Third, we have not confirmed that all mice consumed TJ-41 uniformly. We assumed that the mice consumed almost the same amount of TJ-41 because the food containing TJ-41 was well reduced in the cages, and the body weight of the mice increased gradually.

In conclusion, we demonstrated the anti-inflammatory and systemic condition-improving effects of TJ-41 on COPD mice models. This may be associated with AMPK pathway activation and mTOR pathway inhibition.

## Methods

### Animals

We used male C57BL/6 mice purchased from Jackson Laboratory Japan, Inc., and all mice were reared in a daylight cycle with a defined time from 8:00 AM to 8:00 PM in specific pathogen-free conditions. Male mice were used in this experiment because of the possibility that data may be scattered in female mice due to greater fluctuations in hormonal balance.

**Figure 8:**
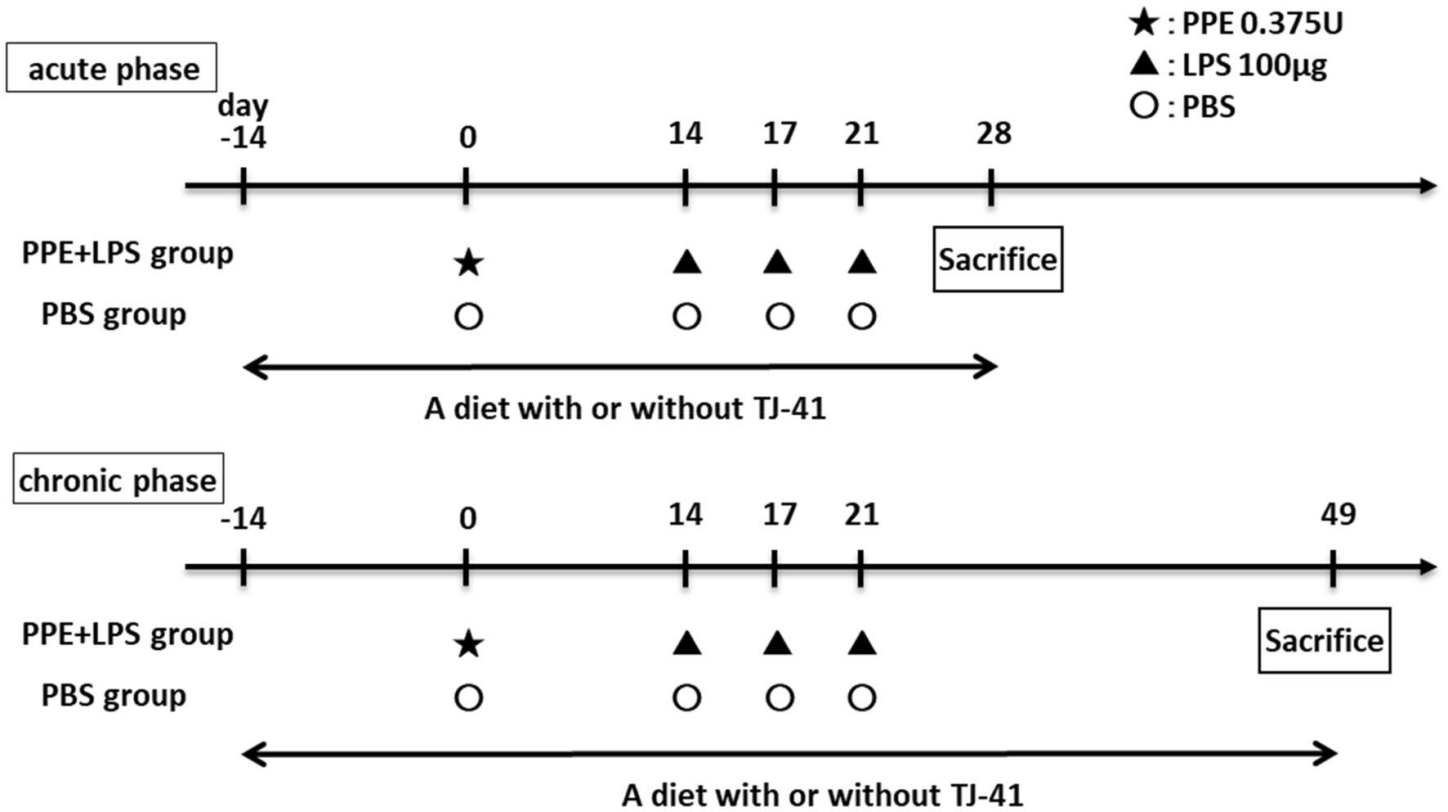
Study design for a COPD mice model. COPD mice model were administered PPE at 10 weeks of age (day 0), and they were treated with LPS three times every 3 days starting at 12 weeks of age (day 14). The 14-week-old mice (day 28) were considered an acute phase model of COPD exacerbation, whereas the 17-week-old mice (day 49) were considered chronic phase models of COPD.

A COPD mouse model was developed by intratracheal PPE administration (E7885; Sigma-Aldrich, USA) followed by LPS (L3024; Sigma-Aldrich, U.S.A.)^25^. At 10 weeks of age (day 0), COPD mice models were intratracheally administered 0.375 U of PPE in 100 µL of PBS under isoflurane anesthesia. At 12 weeks of age (day 14), they were treated with 100 μg of LPS once daily for 3 days. Control mice were administered 100 μL of PBS instead of PPE and LPS (Fig. 8).

A 2% TJ-41-containing or a control diet was fed to mice from 8 weeks of age until physiological and pathological analysis; 14-week-old mice (day 28) were considered the COPD acute exacerbation model, and 17-week-old mice (day 49) were considered the COPD chronic phase model (Fig. 8).

### TJ-41

TJ-41 bulk powder was provided by Tsumura Co. (Tokyo, Japan). Further, 2% TJ-41 was added into rodents’ diets MF (Oriental Yeast Co., Ltd., Japan) for animal experiments, as previously described^24,33^. TJ-41 bulk powder was dissolved in ultrapure water for in vitro experiments. After adding Dulbecco’s Modified Eagle Medium (DMEM, WAKO, Japan) or RPMI-1640 medium (WAKO, Japan) at a ratio of 1:1, the solution was centrifuged, and the supernatant had a final concentration of TJ-41 of 10 μg/μL. The supernatant was added to the culture medium to achieve a final TJ-41 concentration of 50 μg/mL or 250 μg/mL because higher TJ-41 concentrations induced cell death in preliminary studies (data not shown).

### Physiological analysis

After the intraperitoneal administration of combined triadic anesthesia to anesthetize the mice^34^, mice were intratracheally intubated with a blunted 18-gauge needle. It was then connected to SCIREQ flexiVent® (EMKA, Canada) to measure the physiological function of the mouse lung as a closed system. Airway resistance and lung compliance were measured three times, and each average value was adopted.

### Bronchoalveolar lavage fluid analysis

After physiological analysis, 1 mL of sterile saline was injected through the needle using a syringe, the lungs were washed, and the lavage fluid was retrieved three times to obtain bronchoalveolar lavage fluid (BALF). The collected BALF was centrifuged at 450 × g for 10 min at 4℃. The pellet was dissolved in 1 mL of PBS after separating the supernatant, and cells were counted by a LUNA cell counter (Logos Biosystems, Korea). Diff-Quik staining (Sysmex, Japan) was used to measure leukocyte fractions of cells in BALF.

### Lung histopathological analysis

Mouse lungs were fixed by intratracheal injection of 10% buffered formalin solution at a constant pressure of 25 cmH_2_O followed by immersion in formalin solution for at least 24 h. Specimens were embedded in paraffin, cut into 5-μm sections, and stained with hematoxylin–eosin.

### Cell culture

We used two cell types for our in vitro experiments, BEAS-2B cells and U-937 cells, both provided by the Japanese Collection of Research Bioresources Cell Bank. BEAS-2B cells, which is a human epithelial cell line, were grown in DMEM (high glucose) with L-glutamine medium (WAKO, Japan) containing 10% fetal bovine serum (FBS). U-937 cells, which is a human macrophage cell line, were grown in RPMI-1640 with L-glutamine medium (WAKO, Japan) containing 10% FBS. Passages were limited to up to 20 times in each cell.

BEAS-2B cells were seeded in an FBS-free DMEM medium for starvation for in vitro experiments of LPS administration, and TJ-41 was administered at 50 μg/mL or 250 μg/mL. LPS was added at a final concentration of 5 µg/mL after incubation for 24 h. After 2 h, cells were retrieved for mRNA analysis. Starvation was performed in FBS-free RPMI-1640 with L-glutamine medium for U-937 cells, and thereafter, the same procedure was performed for BEAS-2B cells. In vitro studies of LPS administration have reported LPS concentrations of 100–10 μg/mL^35,36^. In particular, 5 μg/mL has been recognized as a highly effective concentration with minimal cell death^37^. Therefore, we decided to administer LPS at a concentration of 5 μg/mL in our study.

BEAS-2B or U-937 cells were starved and treated with TJ-41 solution at a final concentration of 250 μg/mL in time course analysis. The pAMPK, tAMPK, pS6K, and tS6K protein expressions were examined between 5 and 60 min after TJ-41 administration, and Nrf2 and LC3B protein expression was examined between 1 and 6 h after TJ-41 administration.

### Quantitative real-time reverse transcription polymerase chain reaction (RT-PCR)

Cell pellets were lysed with TRIzol (Invitrogen, U.S.A.). Mouse lung tissue was also lysed in TRIzol followed by homogenization using an MS-100 bead homogenizer (Tomy, Japan). After extracting the total RNA, a micro-spectrophotometer was used to confirm that the A260/A280 absorbance of the isolated RNA was > 1.8. From 1.0 µg of total RNA, single-stranded cDNA was synthesized using ReverTraAce (Toyobo, Japan). Quantitative real-time RT-PCR was performed using Thermal Cycler Dice® Real-Time System III and TB Green Fast qPCR Mix (TaKaRa Bio, Japan) according to the manufacturer’s instructions. Quantification was performed in duplicate and the expression levels of target genes were calculated based on *Gapdh* mRNA levels using the delta-delta CT method. Supplementary Table 1 shows the primer sequences.

### Western blotting analysis

Cell pellets were lysed in RIPA buffer (Wako, Japan) with protease inhibitor (Thermo Fisher Scientific, Japan) and phosphatase inhibitor (Thermo Fisher Scientific, Japan). Further, mouse lung tissue was lysed in this buffer followed by homogenization using an MS-100 bead homogenizer (Tomy, Japan). Thereafter, the lysate was centrifuged and the supernatant was collected. The supernatant protein concentration was measured by the BCA method (TaKaRa Bio, Japan), and the protein content of each sample was equalized to SDS (TCI, Japan). After SDS, proteins were electrophoresed, transferred to the membrane, and blocked with ECL prime blocking reagent (GE Healthcare, U.K.). The membrane, as primary antibodies, was incubated overnight in blocking reagent containing pAMPK (50,081; CST, U.S.A.), tAMPK (ab80039; abcam, U.K.), pS6K (9234; CST, U.S.A.), tS6K (2708; CST, U.S.A.), Nrf2 (16,396-I-AP; proteintech, U.S.A.), or LC3B (3868 s; CST, U.S.A.), respectively. The following day, the membrane was incubated for 1 h with the corresponding rabbit IgG antibody (ab6721; abcam, U.K.) or mouse IgG antibody (ab97023; abcam, U.K.), as the secondary antibody. Ez-Capture MG (ATTO, U.S.A.) was used to detect protein bands, and CS Analyzer 3.0 (ATTO, U.S.A.) was utilized to measure band absorbance.

### RNA sequencing

Total RNA was extracted from mice treated with PBS and PPE and LPS using RNeasy Mini Kit (Qiagen, Netherlands) following the manufacturer’s instructions. CLC Genomics Workbench software (Qiagen, Netherlands) was used for RNA sequencing (RNA-seq) analysis. R software was used for statistical calculations, and edgeR software^38^ was used for differential expression analysis. The DEG thresholds were defined as log2 fold change of > 1 or < 1 and a false discovery rate of < 0.05. Gene ontology analysis was performed using the Enrichr combined score^39^. The data set was stored in the Gene Expression Omnibus database (GSE239537).

### Study approval

All mouse experiments were approved by the Ethics Committee for Animal Experiments of The University of Tokyo (P17-006), and were performed in accordance with ARRIVE guidelines and regulations of this committee. In other experiments, all methods were performed according to relevant guidelines and regulations. This study used no human specimens.

### Statistics

Data was presented as means ± standard errors of the mean. GraphPad Prism 5 software (GraphPad Software Inc., USA) was used for statistical analyses. Unpaired *t*-tests were used for the analysis of two groups, and one-way analysis of variance and Tukey’s multiple comparison tests were used for the analysis of three or more groups unless otherwise stated. *P*-values of < 0.05 were considered statistically significant.

### Supplementary Information

Supplementary Information.

## Data Availability

The RNA-seq data generated for this study have been deposited in the GEO repository under accession number GSE239537. The data supporting the results of this study are available from the corresponding author upon reasonable request.
