# Supplementary material for: A Japanese herbal medicine (kampo), hochuekkito (TJ-41), has anti-inflammatory effects on the chronic obstructive pulmonary disease mouse model
Source: Sci Rep. 2024 May 6;14:10361. doi: 10.1038/s41598-024-60646-x (PMC11074295; doi:10.1038/s41598-024-60646-x)
Supplement: Supplementary file 1 — Supplementary Information. [file 41598_2024_60646_MOESM1_ESM.docx]

**A Japanese herbal medicine, Hochuekkito (TJ-41), has anti-inflammatory effects on the chronic obstructive pulmonary disease mouse model**

Masaaki Yuki^1^, Taro Ishimori^1^, Shiho Kono^1^, Saki Nagoshi^1^, Minako Saito^1^, Hideaki Isago^12^, Hiroyuki Tamiya^13^, Kensuke Fukuda^1^, Naoya Miyashita^1^, Takashi Ishii^13^, Hirotaka Matsuzaki^14^, Yoshihisa Hiraishi^1^, Akira Saito^1^, Taisuke Jo^15^, Takahide Nagase^1^, Akihisa Mitani^1^

^1^Department of Respiratory Medicine, The University of Tokyo, 7-3-1 Hongo, Bunkyo-ku Tokyo, 113-8655, Japan.

^2^Department of Clinical Laboratory Medicine, Graduate School of Medicine, The University of Tokyo Hospital, 7-3-1 Hongo, Bunkyo-ku, Tokyo, 113-8655, Japan.

^3^Division for Health Service Promotion, The University of Tokyo, 7-3-1 Hongo, Bunkyo-ku, Tokyo 113-0033, Japan.

^4^Center for Epidemiology and Preventive Medicine, The University of Tokyo Hospital, 7-3-1 Hongo, Bunkyo-ku, Tokyo, 113-8655, Japan.

^5^Department of Health Services Research, The University of Tokyo, 7-3-1 Hongo, Bunkyo-ku Tokyo, 113-8655, Japan.

Akihisa Mitani, E-mail: [mitania5128@gmail.com](mailto:mitania5128@gmail.com)

Corresponding author

**Supplementary Tables**

**Supplementary Table 1.** Mouse RT-PCR primers and human RT-PCR primers.


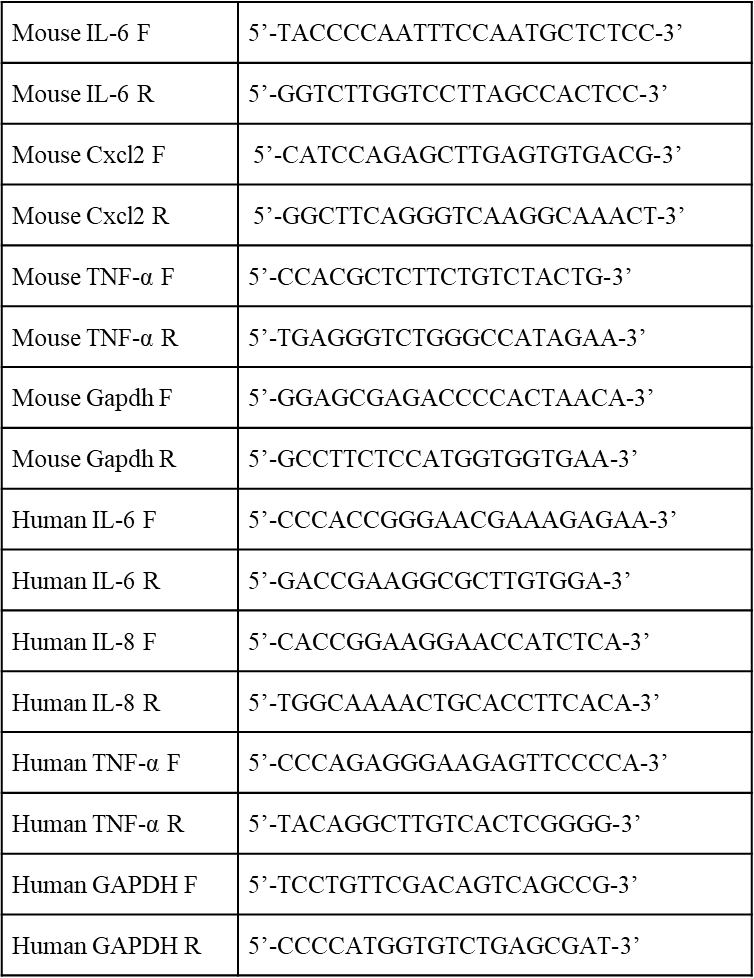


**Supplementary Figures**


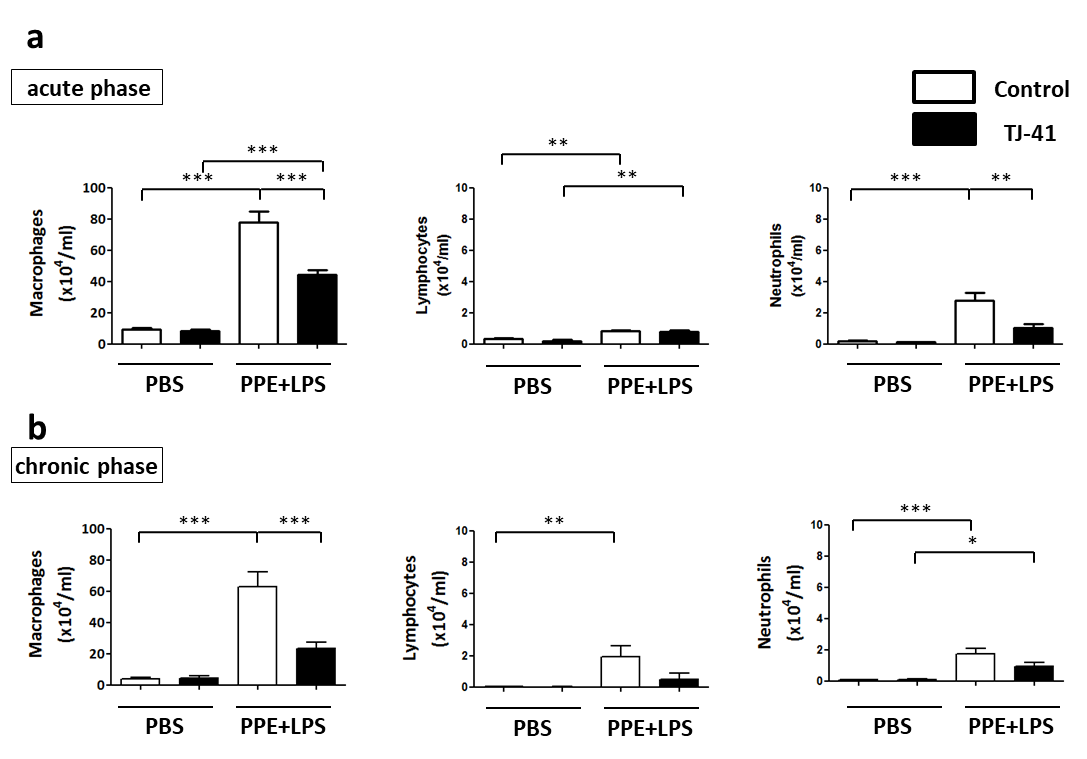


**Supplementary Figure S1.**

Cellular fraction, macrophages, lymphocytes, and neutrophils in BALF were counted in the acute phase (a) (N = 3–5 in each group) and in the chronic phase (b) (N = 7–9 in each group). One-way analysis of variance (ANOVA) test and Tukey’s multiple comparison test, with **P* < 0.05, ***P* < 0.01, and ****P* < 0.001 were considered statistically significant.


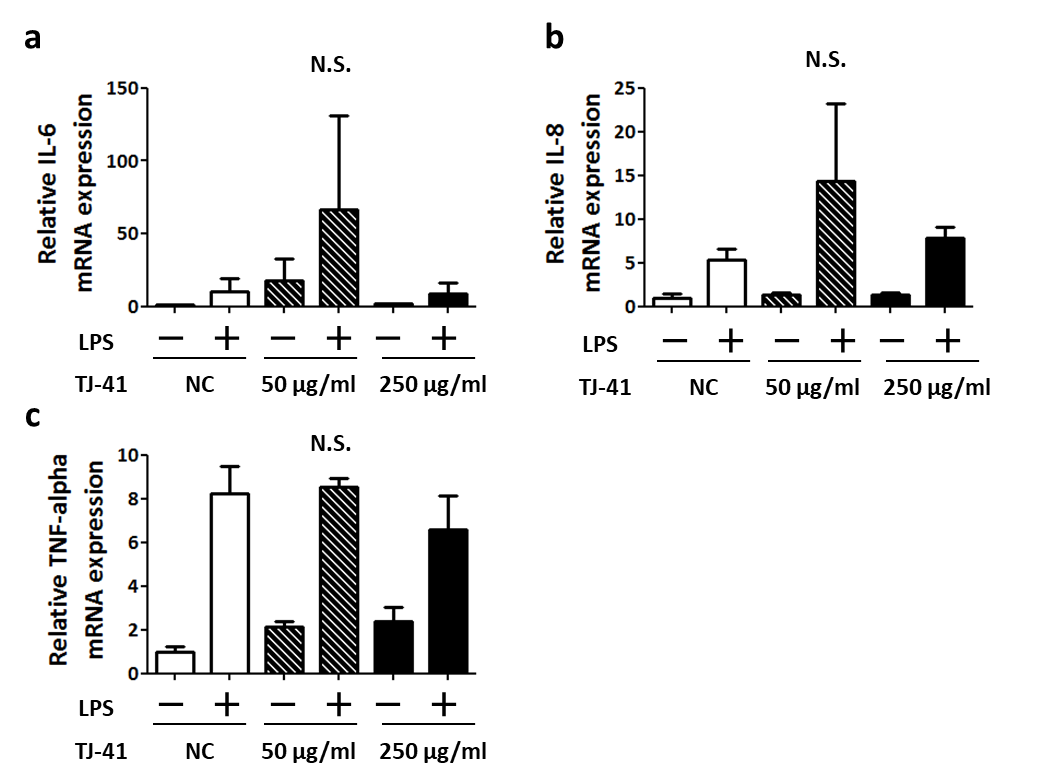


**Supplementary Figure S2.**

mRNA expressions of inflammatory cytokines, IL-6 (a), IL-8 (b), and TNF-α (c), in U-937 cells after LPS treatment with or without TJ-41 administration. None of the differences reached statistical significance in the one-way ANOVA test. N = 3 in each group. NC: negative control; N.S.: not significant.


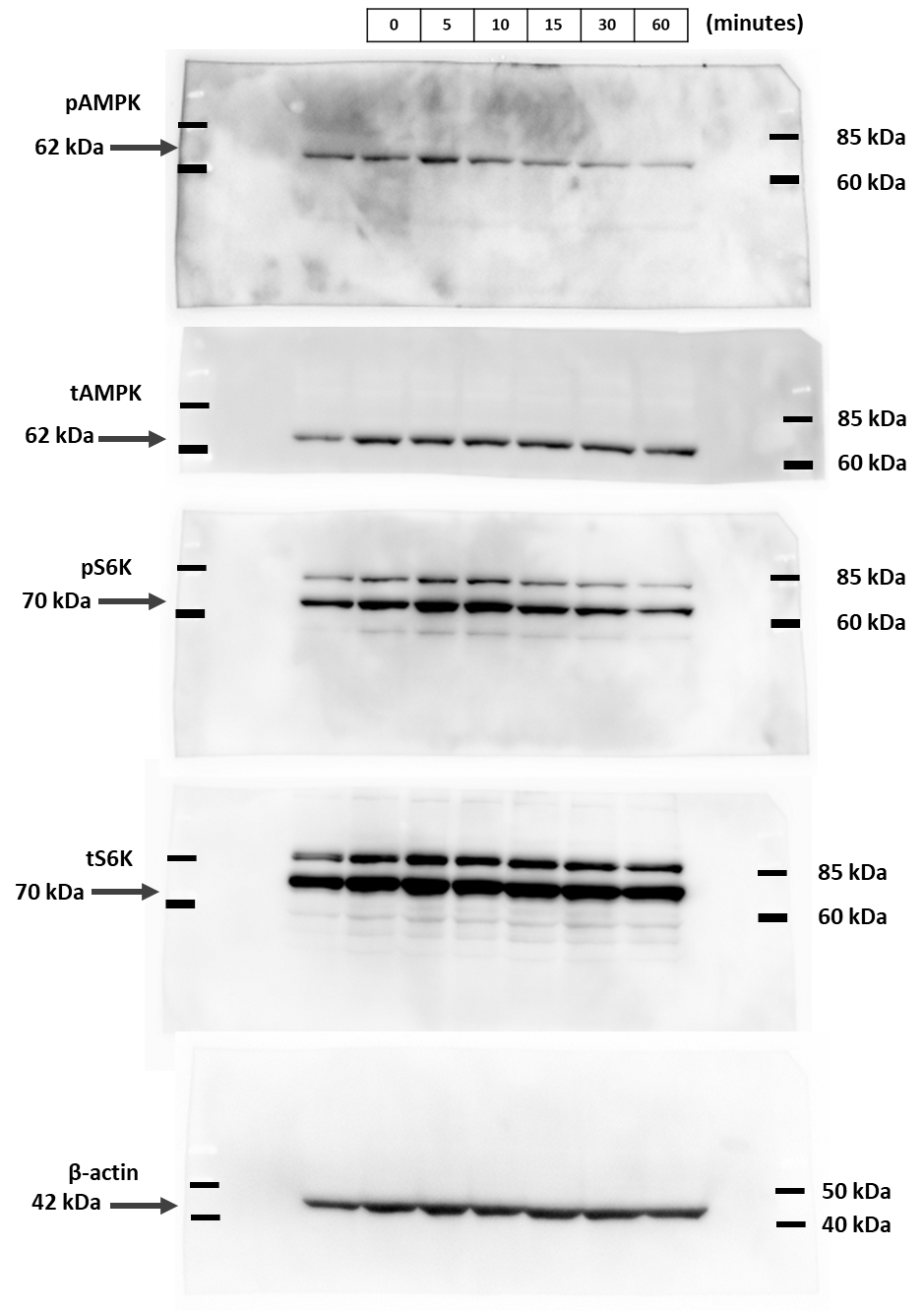


**Supplementary Figure S3.**

Full membrane images for cropped western blotting data from time course analysis between 5 and 60 min in BEAS-2B cells. Additional proteins were loaded in the blank wells of the sides and were not used to calculate the band density. The same membrane was used to detect the protein bands.

**
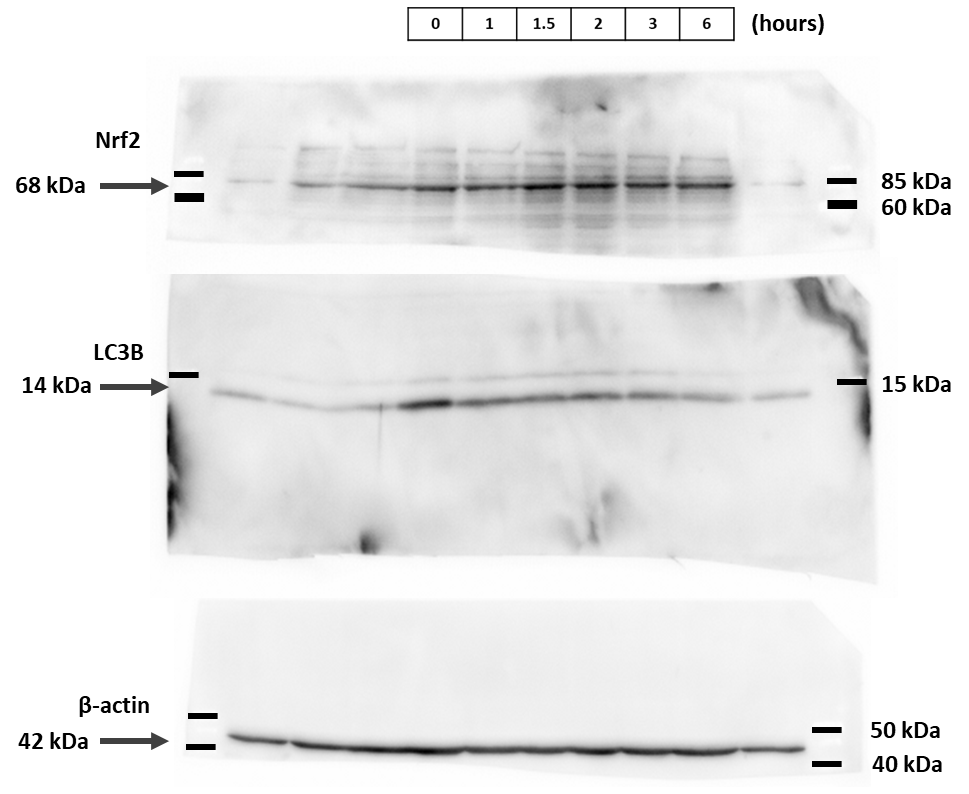
**

**Supplementary Figure S4.**

Full membrane images for cropped western blotting data from long-term analysis for up to 6 h in BEAS-2B cells. Additional proteins were loaded in the blank wells of the sides and were not used to calculate the band density. The same membrane was used to detect the protein bands.


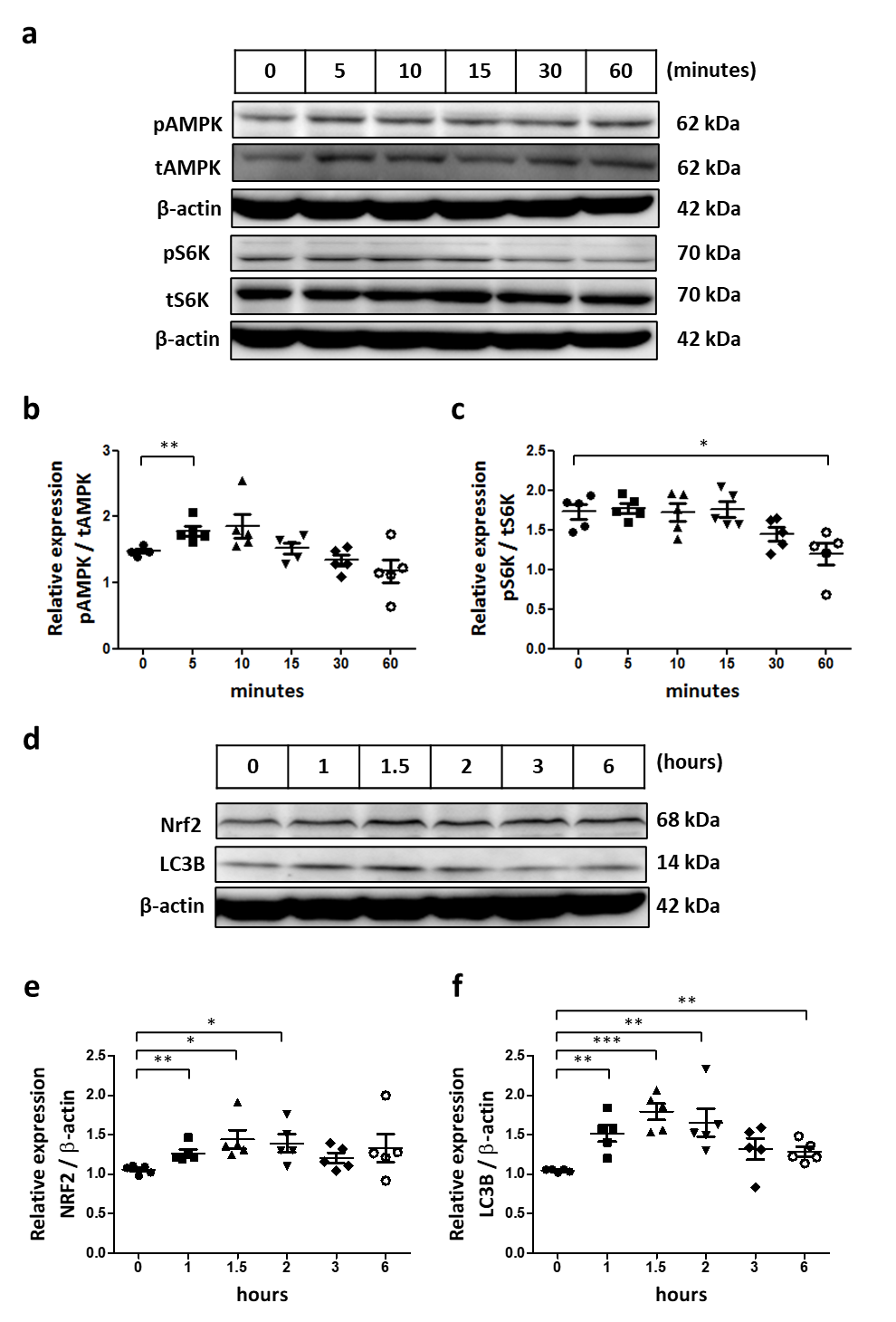


**Supplementary Figure S5.**

(a–c) Western blotting analysis of U-937 cells from 5 to 60 min after TJ-41 treatment. (d–f) Long-time analysis from 1 to 6 h of TJ-41-treated BEAS-2B cells. Unpaired *t*-tests were used for each antibody, and **P* < 0.05, ***P* < 0.01, and ****P* < 0.001 were considered statistically significant. N = 5 in each group.


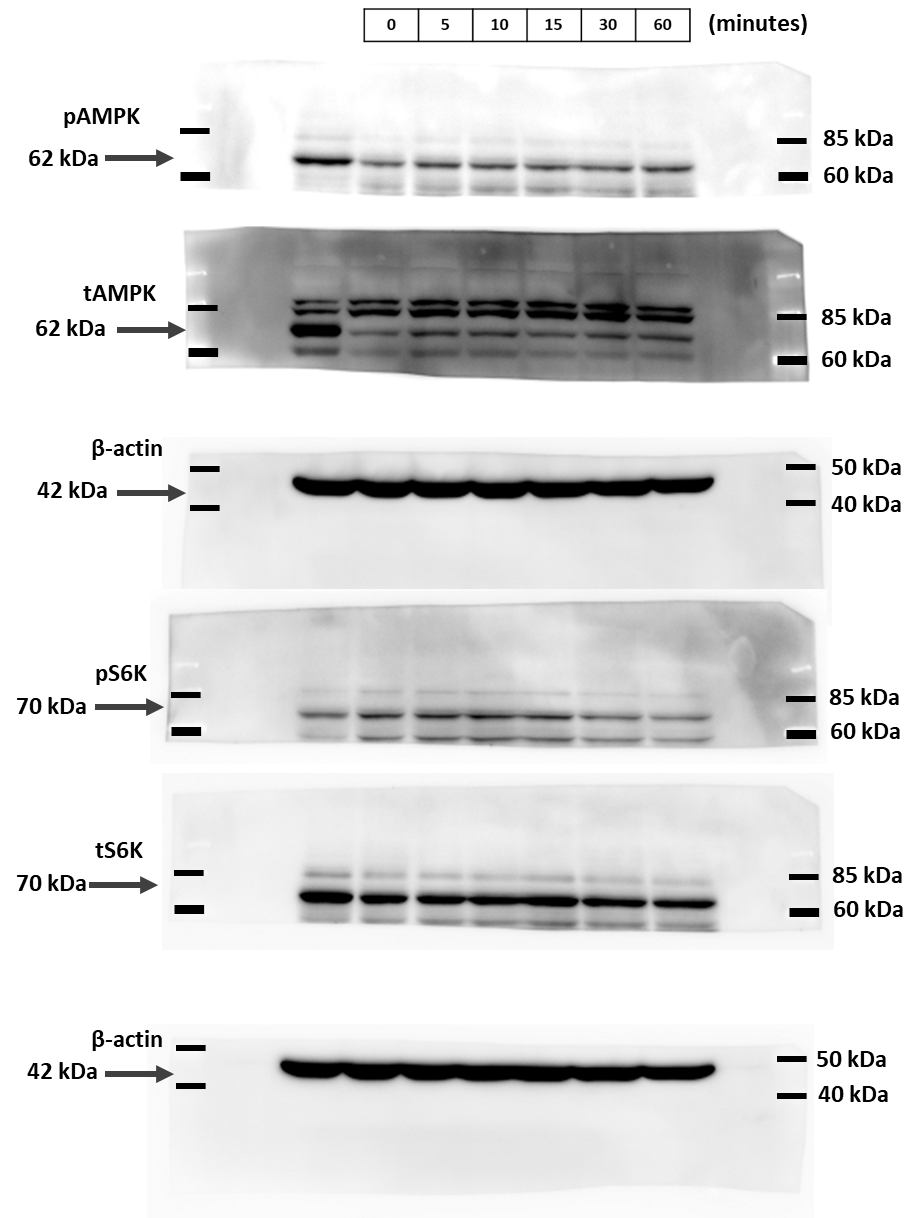


**Supplementary Figure S6.**

Full membrane images for cropped western blotting data from time course analysis between 5 and 60 min in U-937 cells. Additional proteins were loaded in the blank wells of the sides and were not used to calculate the band density. One membrane was used to detect the pAMPK and tAMPK protein bands, whereas the other was used to detect the pS6K and tS6K protein bands.


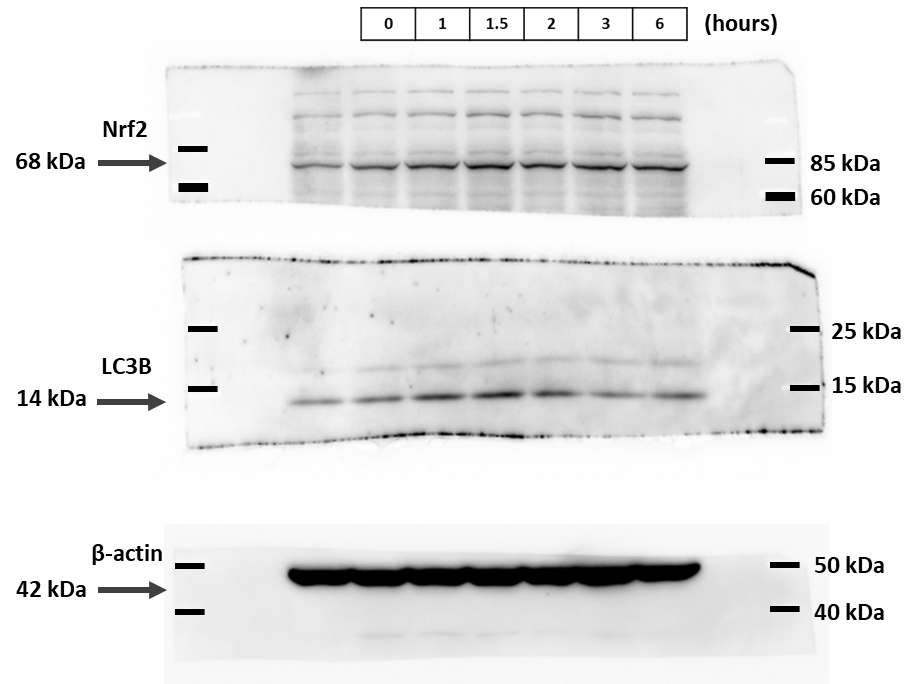


**Supplementary Figure S7.**

Full membrane images for cropped western blotting data from long-term analysis for up to 6 h in U-937 cells. Additional proteins were loaded in the blank wells of the sides and were not used to calculate the band density. The same membrane was used to detect the protein bands.


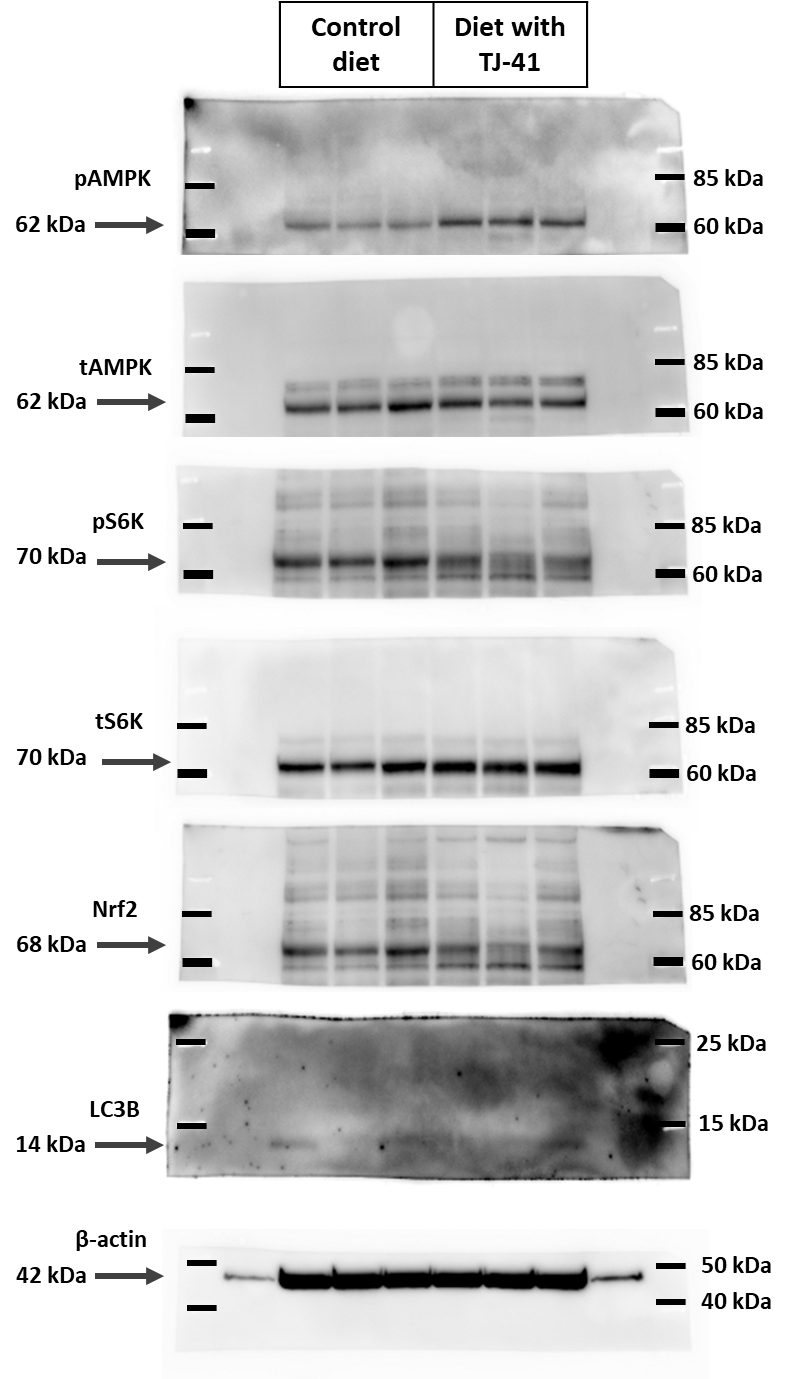


**Supplementary Figure S8.**

Full membrane images for cropped western blotting data from the entire lung tissue of mice fed a control or TJ-41-containing diet. Additional proteins were loaded in the blank wells of the sides and were not used to calculate the band density. The same membrane was used to detect the protein bands.
